# Optimising Use of Electronic Health Records to Describe the Presentation of Rheumatoid Arthritis in Primary Care: A Strategy for Developing Code Lists

**DOI:** 10.1371/journal.pone.0054878

**Published:** 2013-02-22

**Authors:** Amanda Nicholson, Elizabeth Ford, Kevin A. Davies, Helen E. Smith, Greta Rait, A. Rosemary Tate, Irene Petersen, Jackie Cassell

**Affiliations:** 1 Division of Primary Care and Public Health, Brighton and Sussex Medical School, Brighton, United Kingdom; 2 Research Department of Primary Care and Population Health, University College London, London, United Kingdom; 3 Department of Informatics, University of Sussex, Brighton, United Kingdom; University Hospitals of Geneva, Switzerland

## Abstract

**Background:**

Research using electronic health records (EHRs) relies heavily on coded clinical data. Due to variation in coding practices, it can be difficult to aggregate the codes for a condition in order to define cases. This paper describes a methodology to develop ‘indicator markers’ found in patients with early rheumatoid arthritis (RA); these are a broader range of codes which may allow a probabilistic case definition to use in cases where no diagnostic code is yet recorded.

**Methods:**

We examined EHRs of 5,843 patients in the General Practice Research Database, aged ≥30y, with a first coded diagnosis of RA between 2005 and 2008. Lists of indicator markers for RA were developed initially by panels of clinicians drawing up code-lists and then modified based on scrutiny of available data. The prevalence of indicator markers, and their temporal relationship to RA codes, was examined in patients from 3y before to 14d after recorded RA diagnosis.

**Findings:**

Indicator markers were common throughout EHRs of RA patients, with 83.5% having 2 or more markers. 34% of patients received a disease-specific prescription before RA was coded; 42% had a referral to rheumatology, and 63% had a test for rheumatoid factor. 65% had at least one joint symptom or sign recorded and in 44% this was at least 6-months before recorded RA diagnosis.

**Conclusion:**

Indicator markers of RA may be valuable for case definition in cases which do not yet have a diagnostic code. The clinical diagnosis of RA is likely to occur some months before it is coded, shown by markers frequently occurring ≥6 months before recorded diagnosis. It is difficult to differentiate delay in diagnosis from delay in recording. Information concealed in free text may be required for the accurate identification of patients and to assess the quality of care in general practice.

## Introduction

### Electronic health records and their potential for research and clinical audit

Electronic health records (EHR) are a key data source for health-related research [1], [2]. EHR systems are developing rapidly in the US in response to government directives and incentives [3],[4]. EHRs in the UK are most advanced in primary care where they provide complete electronic data about primary care consultations, as well as a summary of care received elsewhere and reported to the practice. EHRs are now seen as valuable resources for research such as healthcare provision audits, disease registries, and epidemiological studies [5],[6]. Traditional epidemiological studies have either used data collected within the study in purpose-built datasets [7],[8],[9] or used routine hospital data which have gone through a separate coding stage (e.g. Hospital Episode Statistics). These can be considered as “static” data, by contrast with “live” electronic health data, produced and coded in the context of clinical consultation and shaped by the context of recording. Many factors influence recording, from financial incentives to the desire to protect a patient from stigma – and these can often change rapidly over time [10]. This means that defining and finding all cases of the disease of interest can be problematic. This paper describes a methodology to maximise the probability of finding cases in EHRs by developing “indicator markers” which are codes that may be combined to make a probabilistic or logical definition of a case in conjunction with, or in the absence of, a diagnostic code. We describe the development of these markers for cases of rheumatoid arthritis (RA) and explore the utility of these markers for case definition in the early presentation of RA.

### The use of indicator markers instead of solely diagnostic codes

When general practitioners (GPs) code the results of clinical consultations, they may not apply a diagnostic code on the first suspicion of a diagnosis, especially of a complex or chronic disease. Instead they may code symptoms, tests or referrals for a specialist opinion. The first firm diagnosis may be found in the text of a specialist's letter to the GP which is attached to the record but coded under an administrative code such as “letter from specialist”. The diagnostic code may only be recorded in the EHR when, for example, a specialised drug prescription is issued. The date of recording of the diagnostic code may therefore bear little relationship to the date of actual diagnosis.

When using EHRs to examine the presentation and diagnosis of a specific condition, it is therefore not sufficient to rely upon the diagnostic codes alone for case definition. Such practice will miss cases for which a diagnostic code has not yet been recorded, and may also misrepresent the date of diagnosis and result in the loss of valuable information about presentation and diagnosis which comes before the code. Two systematic reviews of EHR data quality in the UK [11],[12] found the recording of diagnostic codes for diseases varied, from 40% agreement with other sources for angina, up to 100% for myocardial infarction and diabetes, both of which have clear diagnostic criteria or their recording incentivised in the UK's national health service. Several studies suggest that cancer, for example, is not recorded systematically. Pascoe and colleagues [13] found that when comparing GP records to a cancer registry, 19.3% of patients had no code for a malignancy in their GP records, but that by combining codes for malignancy, abnormal test results and prescriptions they could identify 96.3% of cancer patients in their sample. Tate and colleagues [14] found evidence that a cancer code was recorded late in the diagnostic process, with relevant investigations found in almost three quarters (71%) of patients in the year before the date of recorded diagnosis and 87% having relevant recorded symptoms. In addition, 7.5% had evidence of cancer treatment or oncology referral before any specific mention of cancer as a diagnosis in the EHR.

In order to define cases of the disease of interest in an EHR, it is therefore not sufficient to rely solely on diagnostic codes [15]. Instead it is necessary to draw up lists of a range of related codes, such as symptom, referral, test or treatment codes, which are strongly associated with, and indicative of, the disease of interest. We term these codes “indicator markers”. Indicator markers may be used singly or in combination to indicate the suspicion of, or presumptive presence of, the clinical entity.

### The methodology of drawing up code-lists

Even for studies using only diagnostic codes, the methodology of drawing up code lists for any given clinical entity is poorly described in the literature. Preparing lists for all the various entities that need to be considered within a study – case definition, endpoints, covariates – is a major undertaking. Researchers working on similar diseases in different settings may replicate effort in drawing up code-lists as there is rarely standardisation or transparency to the process. This lack of collaboration means that code-lists and case definitions may vary across studies. Variations in code lists may lead to cases being missed or identified cases differing between studies, and create biased, inaccurate or non-replicable results [15],[16]. Similarly, incidence estimates may be affected leading to inaccurate assessment of the health need, which in turn may have major implications for commissioning and the distribution of resources.

**Figure 1:**
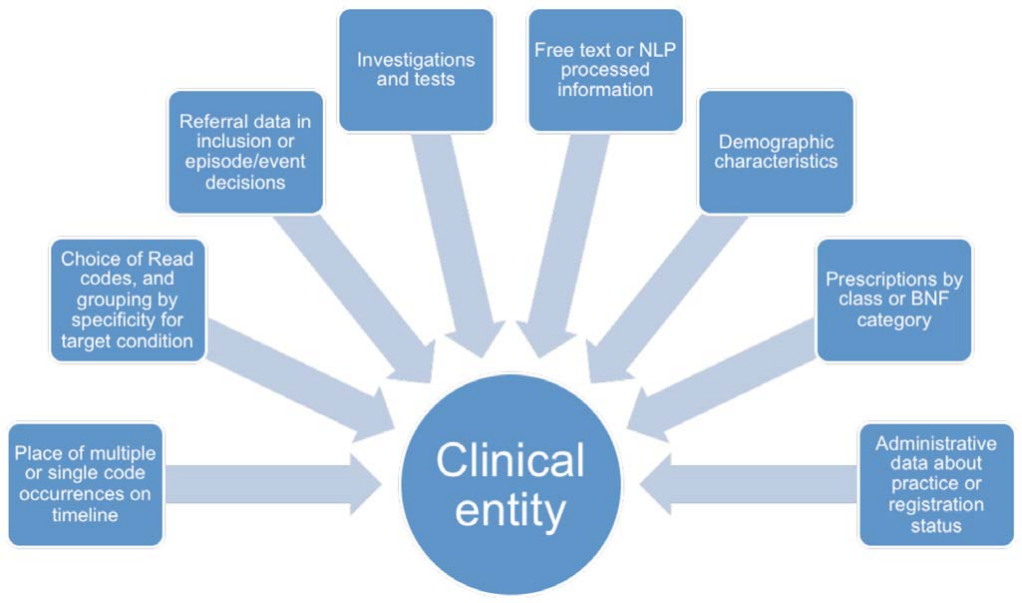
Data types contributing to characterisation of a clinical entity.

Code lists for studies using primary care EHRs are normally prepared through an *a priori* process of consultation between researchers, GPs and disease specialists [17],[18]. A dictionary of Read codes (the hierarchical system for coding diagnoses, symptoms and procedures used in UK primary care) is consulted, and relevant codes selected, which are then operationalised in a case definition. Cases of a disease or “clinical entity” are often defined by the first occurrence of a diagnostic Read code considered as sufficient to define a case (e.g. cancer of the colon). We propose that cases can also be identified by combinations of Read codes and other categories of coded data (e.g. migraine could be ascertained by the occurrence of general terms for headache, together with multiple prescriptions of a migraine specific drug). The multiplicity of potentially relevant data types is shown in Figure 1. A range of these may be combined to form a probabilistic or logical definition. The data types which contribute to case definition of a clinical entity may vary from diagnosis, through symptoms, to consultations and recording of correspondence and may not be confined to the health data of individuals, but may include social, demographic and lifestyle factors. In addition to coded data in the record, much information may be hidden in free text portions of the EHR and this concealed information has very rarely been used to classify cases [19]. Computer based methodologies for reliably extracting written clinical information are still in their early stages [20]. Several authors have shown that including data from free text increases case ascertainment for both acute conditions such as respiratory infections and chronic diseases such as angina [21],[22],[23] as well as RA [19] and can enhance estimates of symptoms in cancer presentation by 40% [24]. Indicators of a disease can be drawn from both coded data and the free text in the record. This study focuses on the use of coded data for this purpose.

### Rheumatoid arthritis as an exemplar

Using descriptions of the early presentation and management of rheumatoid arthritis (RA) in primary care as an exemplar, we aimed to demonstrate the potential impact of supplementing existing approaches to case definition using an *a posteriori*, data driven approach. We hypothesised that if a broad range of indicator markers are to be used to identify cases of RA, they could not be exclusively drawn up using an *a priori* method. Instead, to form a truly comprehensive list of these codes, some scrutiny of data within RA patients' records would be needed. The consequent list of codes developed was then examined for its ability to augment our ability to (i) identify recently incident cases of RA, and (ii) provide information about the diagnostic process in primary care.

Rheumatoid arthritis was selected because robust data on the diagnosis and management of RA in primary care setting is lacking and referral practices are highly variable across the NHS in the UK. However, there is increasing evidence that early intervention with disease modifying anti-rheumatic drugs (DMARD) improves prognosis [25]. Such early intervention requires early recognition and referral to specialist services. Reliable case definition would enable better estimation of presentation and service use as well as facilitate research on this disease. It was hypothesised that there would be significant activity in the record indicative of RA presentation before an RA code was found in the record. RA is not an incentivised condition in UK primary care and there are no universally used codes so identification is problematic. This fact, however, also gave us the opportunity to explore coding in a disease for which there are no agreed coding standards and in which coding practices may vary widely.

### Objectives

Our objectives were to:

1. Draw up a comprehensive list of indicator markers for the early presentation and diagnostic process of rheumatoid arthritis.
2. Describe how much additional information is acquired by supplementing the *a priori* code lists with the *a posteriori* approach.
3. Explore the extent to which the indicator markers could a) be used to identify cases of rheumatoid arthritis prior to an RA diagnostic code and b) describe the diagnostic process of RA in primary care.
4. Explore differences in the recording of indicator markers between genders to confirm that there were no systematic differences between genders which would affect case definition.

## Methods

### Ethics Statement

The study was approved by the MHRA Independent Scientific Advisory Committee (protocol number 09_033R).

### Population and participants

**Figure 2:**
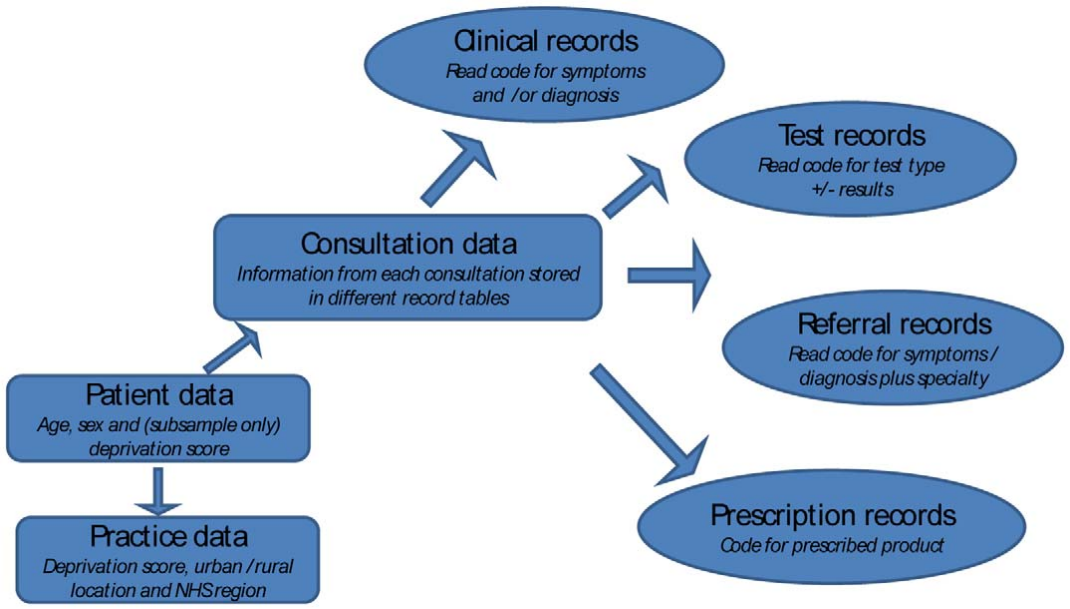
Schematic model of the GPRD database.

The General Practice Research Database (GPRD) is an electronic database of anonymised longitudinal patient records from general practice [1]. Established in 1987, it is a nationwide dataset currently covering 8.5% of the population, with data from over 600 practices, broadly representative of the UK population. There are 5.25 million currently active patients (in January 2012). Records are derived from a widely used GP software system (VISION) and contain complete prescribing and coded diagnostic and clinical information as well as information on tests requested, laboratory results and referrals made at or following on from each consultation. The structure of the data is shown in Figure 2, with different elements of data held in separate record tables. Each clinical event has a code assigned by the GP using the Read coding system, version 2. General practitioners enter medical diagnoses and symptoms using these Read codes. Read codes are a hierarchical recording system used to record clinical summary information. The codes are not limited to diagnostic and procedural codes, but also include codes for symptoms, test results, screening, history and other areas.

Our target population was permanently registered patients in the GPRD within the study period (1/1/2005 to 31/12/2008). From this group we identified patients with a first diagnostic code for RA between 1/1/2005 and 31/12/2008 who were aged 30 years and over at the time of diagnosis.

### Development of indicator markers

**Figure 3:**
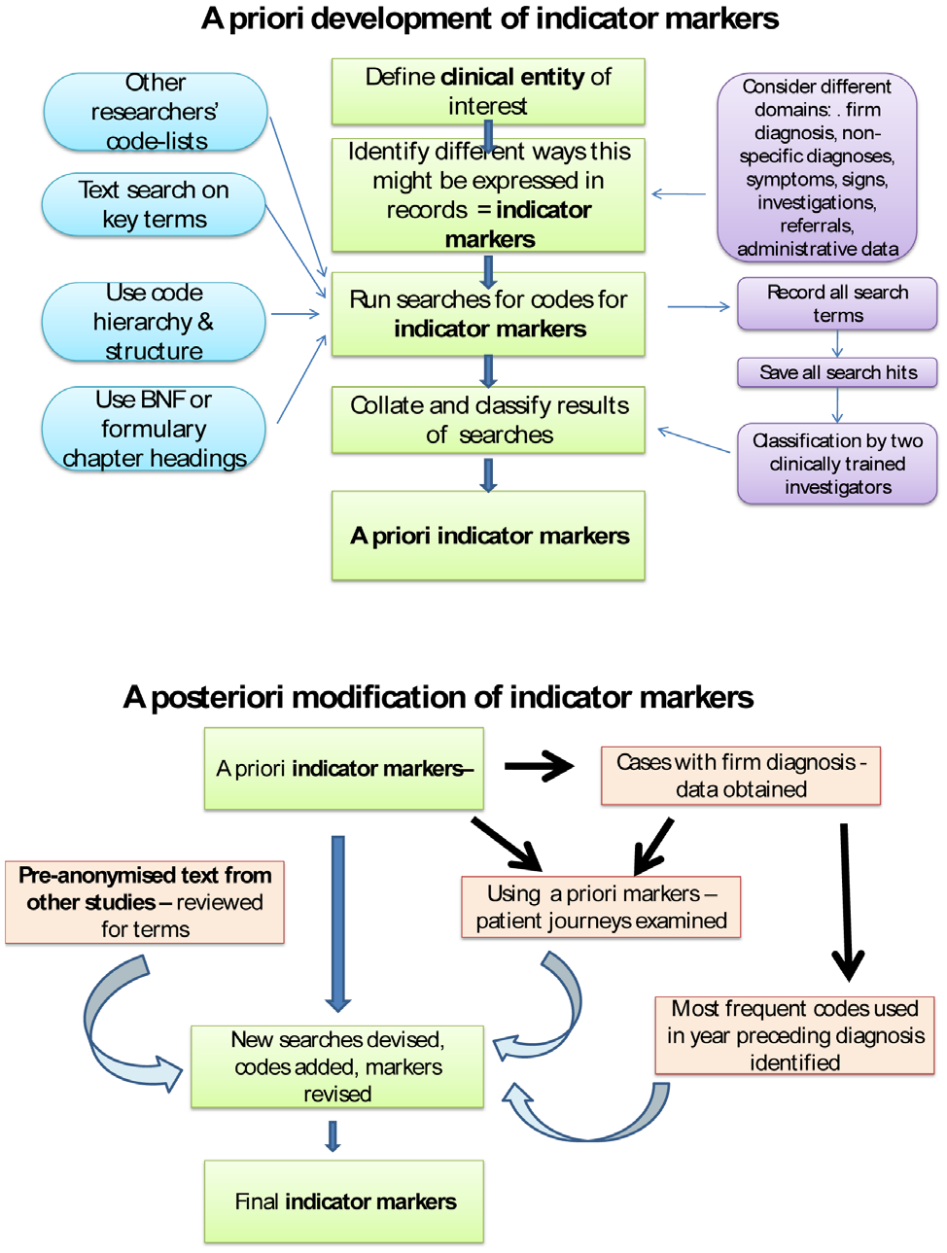
*A priori* and *a posteriori* strategies for developing code lists.

Figure 3 provides an overview of the preparation of the lists of indicator markers. Lists of diagnostic codes for rheumatoid arthritis, used to define the cases used in this study, were also drawn up using the *a priori* methods described here. The methods used two complementary approaches:

1. **a priori** development using clinical opinion, code hierarchy and dictionaries.
2. **a posteriori** modification examining the records of identified RA cases to supplement lists with codes actually used in these records in the period leading up to RA diagnosis.

### A priori development of indicator markers

We were seeking to capture events and consultations involved in the presentation and early management of inflammatory arthritis and which would be recorded in the electronic health record. The lead researcher (AN) met with two rheumatologists (KAD and one other), as well as two GPs (HS, GR) to discuss how patients with early inflammatory arthritis might present and how this might be recorded by the GP in codes in the primary care record. The following domains were considered: diagnoses; symptoms; signs; tests; referrals; treatment. Within each domain, this group of four clinicians identified possible markers of RA presentation along with key terms that might be used to record this presentation (Table 1). Those marked with asterisks were felt to be most specific and were selected as the initial markers.

**Table 1:** Initial list of indicator markers for early inflammatory arthritis across different clinical domains, based on round-table clinical discussions.

Diagnosis	Symptoms	Signs	Investigations	Therapy
* **Rheumatoid arthritis**	*Stiffness especially morning	*Polyarthritis	*Rheumatoid factor	*NSAID
*Inflammatory arthritis	*Painful joints *especially hands/feet/wrists*	*Joint effusion *(not knee)*	C-reactive protein	*DMARD
*Arthritis NOS	Pyrexia of Unknown Origin	Synovitis	ESR (erythrocyte sedimentation rate)	Steroid (non-oral)
*Palindromic RA	Night sweats		Anaemia	Cox II inhibitors
*Reactive arthritis	Fever		X-rays *hand & feet (not knee)*	
Reiter's syndrome			Auto-antibodies	
Fibromyalgia			Thyroid function tests	
Lupus				
Sicca syndrome				

* these markers were selected for initial searches.

We then undertook searches for Read codes used to represent these markers of RA presentation. This was designed to be an inclusive, sensitive search strategy leading to the production of lists of potential codes to be considered. The following combination of techniques was used, often in an iterative fashion.

#### 1. Text searching

This was carried out in the medical browser supplied by the GPRD which lists the codes available to GPs to use in the records. Using the list of keywords that might be used for each marker we searched in as many fields (primarily the Read-Oxmis-Code table) as possible to find suitable codes.

#### 2. Using the code hierarchy

By examining the Read codes obtained from text searches, the main Read code groups involved were identified. Using the NHS Browser, which lists Read codes in hierarchies, related areas in the hierarchy were identified and searches were then repeated including these terms and code-groups.

**Figure 4:**
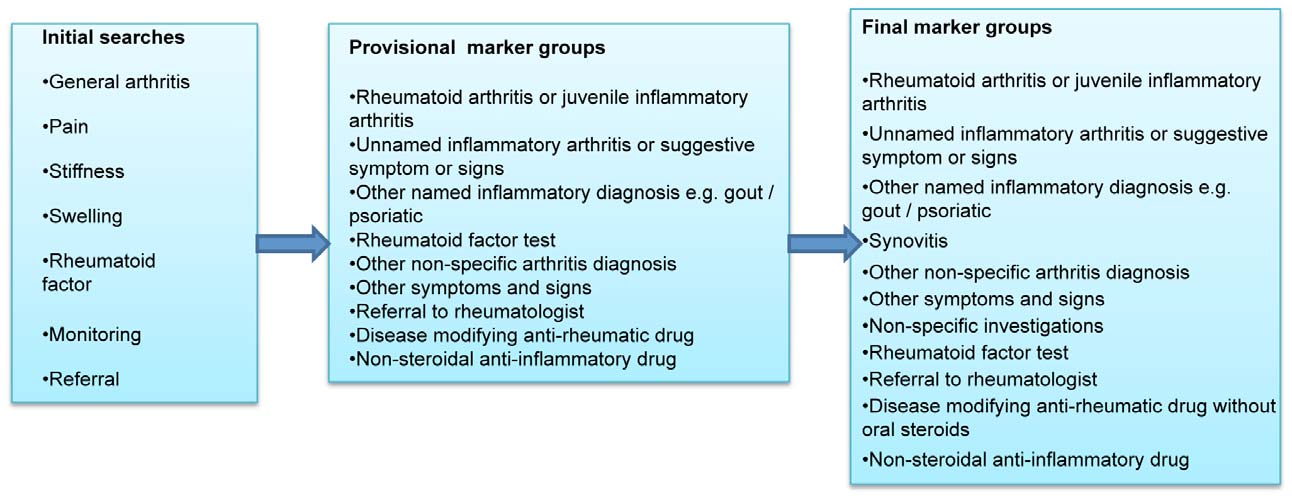
Indicator marker groups.

Figure 4 summarises the marker groups which underpinned the searches that were undertaken. Full details are available in Appendix S1. The search process was complicated by the structure of the Read code hierarchy because the codes for diagnoses and presenting symptoms of musculoskeletal disorders are not clearly differentiated. In order to maintain a complete record of the methods, all codes returned by each search were saved. Code-lists used for studies of RA available from other researchers were reviewed and this led to 15 extra codes being included [15]. Search results were then collated and ten duplicates removed, resulting in a total of 1248 codes relating to markers indicating some aspect of RA presentation. A classification system was prepared so that codes were classified to correspond to provisional indicator marker groups and those that were not relevant to these groups were discarded, with documentation of the process. All codes identified in the searches were examined for relevance and to assign them to marker groups, separately by two clinically trained investigators, AN and either GR or HS. Disagreements were discussed by the coders and if not agreed referred to third party (KD, GR or HS).

### A posteriori modification of indicator markers

We used data from previously identified RA cases in three ways to confirm or modify the *a priori* marker lists drawn up during the first process.

#### 1. Commonly-used codes

The complete records of an initial random sample of 1,743 RA cases defined using the rheumatoid arthritis or juvenile inflammatory arthritis codes in the *a priori* provisional code list were examined. All the codes that related to RA presentation in the year preceding diagnosis were totalled. Any codes that had been used in more than 50 cases but had not been included in the *a priori* marker code lists were reviewed. These codes included a group that described *non-specific investigations* such as C-reactive protein levels, anti-nuclear antibody or other auto-antibody tests. These were added as new marker group. Additional symptom codes for knee pain and neck pain were added to the *other symptoms* marker and polymyalgia rheumatica was added to the *other named inflammatory arthritis diagnosis* marker.

#### 2. Patient histories

For selected time periods all the codes used in 30 randomly selected RA patients from the sample above were reviewed. The time periods selected were 30 days either side of first *referral* code, first *Disease Modifying Anti-Rheumatic Drug (DMARD)* prescription (especially if more than six months before diagnosis), and 30 days either side of the first one out of *inflammatory diagnosis*, *rheumatoid factor test*, and *non – specific investigation* code (especially if these were more than six months before diagnosis). This review revealed that one code for *psoriatic arthropathy* had been missed. It additionally suggested that many oral steroid prescriptions were unrelated to arthritis, so the codes for DMARD were revised to remove *steroids*. It also revealed use of codes for *monitoring DMARD usage*. New searches were undertaken for codes related to *drug monitoring*, the results were classified by two coders and the agreed codes added to the DMARD code-list.

#### 3. Pre-anonymised text

We had access to 10,000 entries of pre-anonymised text from the GPRD unrelated to this study dataset. These had been used in a variety of other studies, including an investigation into the use of non-steroidal anti-inflammatory drugs. The free text entered in association with 1307 codes which were one of the assigned *a priori* codes or had the term *arthritis* in the text was reviewed. Terms in text that might be useful in identifying cases were added and new searches performed (as in the *a priori* code – searching process) and classified using methods described above. This led to a marker of *synovitis* being created (Figure 4).

Final code-lists for the indicator markers are in Appendix S2. Markers were separated into *specific markers* of rheumatoid arthritis (inflammatory arthritis, other inflammatory condition, referral to rheumatology, rheumatoid factor test, synovitis or DMARD prescription) and *non-specific markers* (non-specific or other arthritis diagnosis, non-specific inflammatory or auto-immune investigations, joint signs and symptoms and NSAID prescriptions). No changes were made to the diagnostic codes for Rheumatoid Arthritis or Juvenile Inflammatory Arthritis in this *a posteriori* modification process.

### Identification of cases

All patients with a first coded diagnosis of RA (from an *a priori* list of diagnostic codes) and aged 30 years or more at the time of diagnosis were identified in the study period (2005–2008). Patients who had an RA code anywhere in their record before the study period were not included. GPRD allows for a date to be entered for an event, in case this differs from the date the event was recorded. However, if an event date had not been entered into the GP system, the date that the record was created was used (10,986 events (0.1%) were imputed). Events were discarded if they occurred before the start date (the later of the patient's registration date or the date that the practice's records were considered up-to-standard by the GPRD) or after the end date (the earlier of the date of the patient leaving the practice or the last date records were received from the practice). The indicator markers (asterisked in Table 1) were identified in the records within one year of RA diagnosis. Having established the markers, we then sought these codes throughout the records of RA patients. To standardise follow up times, analyses were restricted to RA cases who had records for at least three years before through to 14 days after the first coded RA diagnosis. All records were cut off at three years prior to diagnosis. The first diagnostic code had to be within the study period (2005–2008) but indicator marker events occurring before the study period were included in analyses.

### Statistical analysis

The data were prepared using Stata version 11 (Statacorp LP, Texas). For each indicator marker, any relevant code from any GPRD entry resulted in a positive marker. Within a marker group, the date of the earliest code was taken as the first example of an indicator for that patient and the date intervals were calculated from this earliest code. For medication markers (DMARD and NSAID) we looked for the presence of a prescription of these medications. The prevalence of different indicator markers was compared using chi-squared tests. The time interval between the first indicator marker within a group and the first coded diagnosis of RA was calculated. Since the time-intervals were skewed, medians and non-parametric tests were used to compare groups (Mann Whitney U). Survival analysis was performed constructing survival time from 14 days after diagnosis backwards to three years, with the earliest code in any indicator marker group as a failure event.

## Results

Results describe the value of the addition of indicator markers to diagnostic code case definitions and how their exploration changes the view of RA management in primary care.

### Cases defined by diagnostic codes

Cases were defined by a first occurrence of an RA diagnostic code during the study period (and no code prior to this in their entire record). The study sample comprised 5,843 cases of RA, of which 1,831 were men and 4,012 were women. Men were older (median age 63 years; inter-quartile range, (IQR) 51–73) than women at the time of diagnosis (median age 60 years; IQR 50–71, p<0.001).

### Prevalence of Indicator Markers

Table 2 summarises the prevalence of the indicator marker groups both in the whole study period and within particular time periods. The most common **specific marker** was a rheumatoid factor test, for which 62.9% patients had a code. There were codes for referral to rheumatology services in 42.4% of patients. Codes for clinical findings related to any inflammatory arthritis were uncommon, present in only 14.8% of patients. Likewise synovitis codes were present in only 4.1%. A third (33.6%) of patients had a prescription for a DMARD and in 14% of patients this had occurred *at least six months before* the diagnosis of RA was coded. Overall, 86.0% of patients had one or more specific indicator markers including DMARD prescriptions, with 34.2% having one marker, 30.5% having two markers and 21.3% having three or more specific markers.

**Table 2:** Prevalence of indicator markers during study period.

Marker present		Timing of first marker within study period (in relation to RA diagnosis)			
		14 days after to <1 month before	1–3 months before	3–6 months before	6 months – 3 yrs before
**Non–specific inflammatory arthritis diagnosis or signs**					
**n**	867	209	143	124	391
**%**	14.8	3.6	2.5	2.1	6.7
**Other named inflammatory condition**					
**n**	410	32	32	49	297
**%**	7.0	0.6	0.6	0.8	5.1
**Referral to rheumatology services**					
**n**	2,480	725	467	306	982
**%**	42.4	12.4	8.0	5.2	16.8
**RhF test – regardless of result**					
**n**	3,674	1326	657	488	1,203
**%**	62.9	22.7	11.2	8.4	20.6
**Synovitis**					
**n**	241	46	44	33	118
**%**	4.1	0.8	0.8	0.6	2
**DMARD prescription – excluding oral steroids**					
**n**	1,962	842	154	123	843
**%**	33.6	14.4	2.6	2.1	14.4
**Any specific marker (date of earliest marker used)**					
**n**	2057	1395	771	607	2243
**%**	86.0	27.8	15.4	12.1	44.7
**Non-specific arthritis diagnosis**					
**n**	1,053	200	224	151	478
**%**	18.0	3.4	3.8	2.6	8.2
**Joint signs & symptoms**					
**n**	3,823	363	440	421	2,599
**%**	65.4	6.2	7.5	7.2	44.5
**Non–specific investigations e.g. CRP, auto-antibodies**					
**n**	3,861	844	611	519	1,887
**%**	66.1	14.4	10.5	8.9	32.3
**NSAID prescription**					
**n**	4,631	385	390	373	3,483
**%**	79.3	6.6	6.7	6.4	59.6
**Any non-specific Markers (date of earliest marker used)**					
**n**	5524	404	370	400	4318
**%**	94.5	7.4	6.7	7.3	78.6

#### Non-specific markers

were more common in the records but these may reflect activity relating to events other than RA. The most common indicator marker was a prescription for a non-steroidal anti-inflammatory drug (NSAID) which was present in over three-quarters of patients (79.3%). Two-thirds of patients (66.1%) had evidence of a non-specific investigation, such as CRP or autoantibody level and two-thirds (65.4%) had evidence of other joint signs and symptoms. Non-specific arthritis diagnoses were found in 18.0% of patients. Overall 94.5% of patients had one or more non-specific indicator markers. When NSAIDs were excluded from this analysis, as they may have been prescribed for many conditions other than RA, 86.0% of patients had at least one non-specific marker, with 32.8% having one, 42.8% having two, and 10.4% having three non-specific markers.

In this sample, only 4.9% of patients (288) had no indicator markers whether specific or non-specific. Only 11.5% (674) patients had a single indicator marker, whereas 18.3% had two markers, 23.6% had three markers, 21.1% had four markers and 20.5% of patients had five or more indicator markers in their record before the RA diagnostic code was recorded.

### Combinations of Markers

The combinations of specific indicator markers were examined to assess their utility in presumptive case definition. These are shown in Table 3. The most common specific indicator markers (DMARD prescription, referral to rheumatology and rheumatoid factor test) were assessed in combination with each other, as well as with joint signs & symptoms from the non-specific markers.

**Table 3:** Frequency of combinations of indicator markers.

Code 1	Code 2	N (5843)	%
DMARD	Referral	1175	20.1
	RhF Test	1032	17.7
	Joint Symptom	1263	21.6
Referral	RhF Test	1640	28.1
	Joint Symptom	1764	30.2
RhF Test	Joint Symptom	2670	45.7
DMARD, referral and RhF Test			
- any two		1846	31.6
- all three		667	11.4
DMARD, referral, RhF Test and Joint symptom			
- any two		2080	35.6
- any three		1426	24.4
- all four		531	9.1

#### DMARD prescription

This was commonly found in combination with other markers: 1175 patients (20.1%) had both a DMARD prescription and a referral to rheumatology services before their RA code, 1032 (17.7%) had both DMARD and a rheumatoid factor test and 1263 (21.6%) of patients had DMARD and a non-specific joint sign or symptom.

#### Referral

1640 patients (28.1%) had both a referral to rheumatology services and a rheumatoid factor test coded in their file before the RA code was recorded and a similar number (1764; 30.2%) had both a referral and a joint sign or symptom.

#### Rheumatoid factor test

Nearly half of patients (2670; 45.7%) had codes for both a rheumatoid factor test and a joint sign or symptom.

When the three most common specific markers were investigated (DMARD prescription, referral and rheumatoid factor test), it was found that 1846 patients (31.6%) had two of these markers and 667 patients (11.4%) had all three of these markers in their file before an RA code was recorded. When joint signs & symptoms was added to this list, 2080 patients (35.6%) had two of these markers, 1426 patients (24.4%) had three markers and 531 (9.1%) had all four of these common markers.

### Prevalence of Markers by Gender

The prevalence of markers was similar in men and women as shown in Table 4. Only two marker groups showed statistically significant gender differences in prevalence. Diagnosis of a named inflammatory arthritis was more common in men, 8.3% compared to 6.5% in women (chi square  = 6.18, DF  = 1; p = 0.01) (not shown in table). A DMARD prescription was also more frequent in men, 35.7% compared to 32.6% in women, (chi square  = 5.47, DF  = 1; p = 0.02), although when a Bonferroni correction was applied for multiple comparisons, these differences were no longer significant.

**Table 4:** Intervals between first indicator marker and diagnosis in RA cases (days).

	n	%	Mean	Median	25% quartile	75%% quartile	n	%	Mean	Median	25% quartile	75%% quartile	p for gender difference*
**Inflammatory **arthritis****diagnosis	273	14.9	278	126	34	454	594	14.8	307	159	34	511	0.31
**Rheumatoid **factor test	1,142	62.4	193	74	13	250	2,532	63.1	212	75	9	303	0.94
**Referral**	801	43.7	233	88	16	326	1,679	41.8	269	109	22	445	0.02
**DMARD **prescription	654	35.7	321	51	0	693	1,308	32.6	351	103	0	749	0.23
**Non-specific **arthritis****diagnosis	351	19.2	258	141	44	370	702	17.5	304	154	45	508	0.19
**Non-specific auto-**immune****test	1,198	65.4	295	147	38	478	2,663	66.4	336	189	42	588	0.006
**Joint signs and **symptoms	1,186	64.8	424	323	94	747	2,637	65.7	481	448	129	813	<0.001
**NSAID **prescription	1,455	79.5	577	595	160	1010	3,176	79.2	614	689	194	1021	0.02

* non-parametric Mann-Whitney U test.

### Timing of Markers

The timings of the first occurrence of the indicator markers in relation to RA diagnostic code are shown in Table 2 and Table 4. Referral to rheumatology services, rheumatoid factor tests and DMARD prescriptions were coded closest in time to diagnosis, whereas non-specific markers such as general investigations or symptoms were frequently present more than a year before diagnosis. It was found that 20.6% of patients had a rheumatoid factor test coded more than six months before a specific diagnostic code was recorded, 16.8% had a referral to rheumatology and 14.4% had a DMARD prescription. Overall, 44.7% of patients had a specific marker occurring more than six months before a diagnostic code was recorded. Intervals between markers and RA diagnostic code were longer in women than men for non-specific auto-immune type investigations (men 147 days (median), women 189 days; p = 0.006), for presentation of joint signs and symptoms (men 323 days (median), women 448 days; p<0.001), and for first evidence of referral (men 88 days (median), women 109 days; p = 0.02).

**Figure 5:**
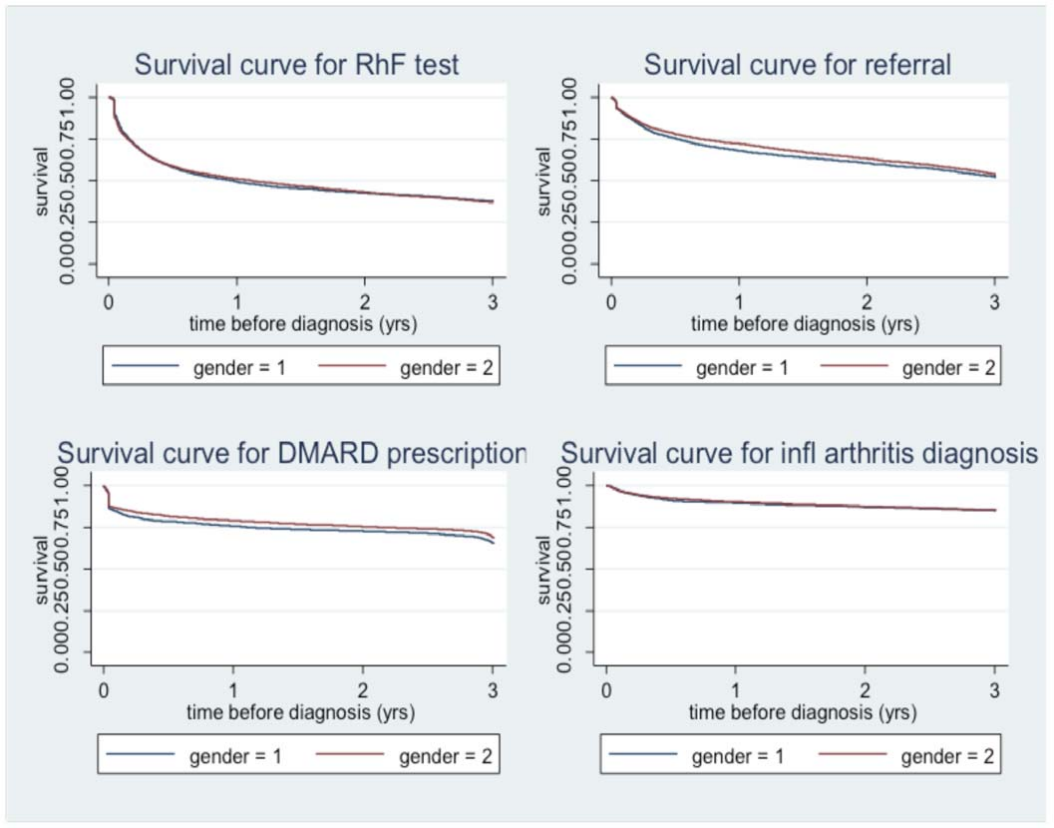
Survival curves for selected indicator markers in the 3 **years preceding RA diagnosis, by gender.**

Figure 5 shows “survival” curves for selected indicator markers, with time reversed, from 15 days after diagnosis to three years before diagnosis with a “failure event” being the earliest marker in that indicator category. The curves show that rheumatoid factor tests, referral and DMARD most often occur close to diagnosis with a more gradual occurrence more than one year previously.

## Discussion

This study described in detail a two stage, iterative process for drawing up code lists and looked at the utility of indicator markers prior to the definitive diagnostic code in a) the description of early care, and b) the case definition, of rheumatoid arthritis patients, with a view to defining uncoded cases (false negatives). Following a panel of clinicians drawing up a first list of codes, data from RA patients was scrutinised to see if additions to the lists should be made. The second stage of this process resulted in considerable modification of the lists, suggesting this is a valuable addition to traditional methods of approaching code list design. The indicator markers chosen were found to exist in the file well before the RA diagnostic code and to give important information on the diagnostic process of RA. Indicator markers were wide-spread in the records of RA patients with 83.5% of patients having at least two markers in their file before the RA diagnostic code was recorded.

This strategy of drawing up code lists, with the combination of *a priori* and *a posteriori* data-driven stages, will be applicable to other conditions and coding systems. Using the second stage, *a posteriori* approach allowed us to make significant modifications to code lists. For example, synovitis and non-specific auto-immune investigations had been raised by the expert group at the initial stages but not included in the *a priori* searches as they were considered too non-specific. However, they were discovered, during the data-driven stage, to be found with some regularity in the records of RA patients. The data-driven stage then revealed codes that had been missed and some errors in classification and so served as a form of triangulation. The finding that this data-driven modification stage is of such importance poses a challenge to researchers and data providers, as this additional stage of code searching may not be possible in the design stage of all research studies, due to difficulties with advance access to data.

Secondly, our study looked at the value of using indicator markers to understand the diagnostic process of early RA as presenting in primary care. Using these markers, we aimed to indicate locations in the EHR where the diagnosis of RA was being considered and to describe the early course of disease presentation in these patients, as shown by coded symptoms of the disease, referrals to appropriate services or because of tests being ordered or results recorded. Indicator markers were widespread within the RA patient records, in many cases a long time before the RA diagnostic code was found on the file. These results suggest, as was hypothesised, that the diagnosis of RA may have been known about for some time before a diagnostic code was added, with specific markers frequently found more than six months before recorded diagnosis. This discrepancy between dates may indicate diagnostic uncertainty or delay in coding a known or presumptive diagnosis. Further field work would be needed to determine which of these two processes has most influence in these data. UK guidance from NICE [25] suggests that RA should only be diagnosed after it has been in evidence for 6 months. However, these data precede this guidance and this is unlikely to be a full explanation for the delay in diagnostic coding.

We were also able to explore gender differences in indicator markers, to check for systematic differences in recording by gender, but found no evidence of gender differences in the prevalence of most, except for named inflammatory arthritis and DMARD prescriptions both of which were more common in men. The prevalence of referral and investigation markers was similar in men and women although there was some evidence that the gap between referral, non-specific symptoms, non-specific investigations and coding of diagnosis was longer in women than in men.

Thirdly, this study explored indicator markers as a way of broadening case definitions in this disease, thereby potentially identifying individuals with the disease who do not yet have a diagnostic code. The majority of patients (83.5%) had two or more indicator markers, and therefore combinations of codes may help to describe the early presentation and diagnostic process of these patients. One finding, however, was that the most common symptom codes found in our study were the non-specific ones, and these could relate to conditions other than RA. Despite this, 86% of patients had at least one specific marker. When a combination of the four most indicative markers were examined, it was found that 36% had two of these markers, 24.4% had three markers and 9.1% had all four of these common markers. This suggests that using these markers in combination may facilitate the identification of RA cases, both in the early stages before an RA code is recorded and in cases where no code has been recorded and the cases would ordinarily slip “under the radar”, leading to false negatives and biasing a subsequent study.

However, these findings suggest that while coded indicator markers do give a good indication of the presentation of RA, in 16.5% of patients there was no marker or only one marker before the RA diagnostic code. With coded information alone it may not be possible to reliably identify the early presentation of every case. Extra information may be available in unstructured free text, and may include both notes made by the GP during a consultation and the content of referral letters and correspondence from specialists. Information governance requirements mean that free text must be anonymised before release to researchers. It is not currently possible to do this reliably automatically and therefore manual, expensive labour is required to ensure that datasets are anonymised. Research projects using EHRs have therefore relied almost exclusively on coded structured data rather than accessing free text [12]. Text data is also difficult to analyse in large numbers of patients and requires some form of processing or structuring to allow quantitative analysis [13]. The supplementation of coded information with free text would significantly strengthen findings in studies using EHRs, as has been found in previous studies of case definition of RA using EHRs [19],[26].

In order to identify a “case” for a study using EHRs we therefore propose that there are three levels of classification for sources of variation in recording, each level posing an increasing challenge to the researcher. **Level 1** consists of diagnostic codes from within the same domain, such as different diagnostic codes for rheumatoid arthritis. Because these are diagnostic codes, it is probable that patients with these codes have the condition of interest. However, the sole use of these codes may miss cases where diagnostic coding is delayed or incomplete. At **level 2**, codes from different domains are used, such as symptoms or test results rather than a diagnosis e.g. “joint stiffness”, “referral to rheumatology clinic” or “rheumatoid factor positive”. A combination of these codes would result in a probabilistic definition of the condition, but these coding patterns result in less certainty in the diagnosis for the researcher and may also result in the inclusion of cases which do not have the disease of interest. The final level, **level 3**, is where additional disease specific information is found in free text – either along with a more general diagnostic code or a code for a symptom or a more general code such as “had a chat to patient”. Free text may supply additional information to allow a diagnosis to be made with more certainty than if coded data alone were used.

### How findings fit with previous literature

Literature on the methodology of drawing up code-lists for the identification of cases, treatment or outcomes for health research is sparse, despite its pivotal importance in such research. Some work been published on the operationalisation of code-lists into statistical programmes [27]. There are techniques available to address the proliferation of diagnostic codes such as ancestor/descendant tracing within SNOMED (“Systematised Nomenclature of Medicine Clinical Terms” – an international comprehensive terminology system being adopted by the NHS), hierarchies within Read codes and query building within clinical trial systems. However, high-level search strategies, and how to address variations in lists of codes below Level 1 variation (outlined above) have not been discussed in the literature.

A lack of standardisation in methodology means that code-lists, and hence case definitions, may vary across studies in a variety of research contexts. Very little is known about how this variation in code-lists affects study results, although there are implications that it may be important, such as high rates of false negatives or positives introducing biases in the estimation of uptake of certain tests or treatments within disease populations [16],[28]. An additional concern is that the use and type of coding structures may be individual to software systems so that the codes used to describe, for example a polyarthritis, may differ across different primary care software systems. Such system-specific coding reinforces the need for a data-driven stage in any case-finding process and the need to explore the data before finalizing code selections.

Other authors have also highlighted the deficits from coded data [29]. Jordan and colleagues showed that the number of consultations related to knee pain were underestimated without the use of text from primary care databases [21], and as mentioned Tate and colleagues have shown that the diagnosis date of ovarian cancer recorded in primary care records is later than other evidence in the record [14]. This is consistent with our results indicating that the diagnosis may have been known for some time before it was formally coded. In addition two studies have found that using free text within algorithms for case definition significantly improves the positive predictive value of subject classification in RA when using EHRs [19],[26].

Previous studies looking at the presentation and management of early RA have been situated in secondary care and are therefore prone to substantial referral bias [30],[31],[32]. The existing work suggests that delay in seeking medical attention is a more important factor than delay in recognition and referral to specialist services [33]. Data from primary care describing the care process from the first presentation are needed, but our results suggest that this cannot be achieved within routine primary care electronic databases using codes alone as much information, particularly that which occurs outside primary care, may be recorded in free text.

### Limitations

The indicator markers have not been validated by a review of the associated text in the record and therefore it is possible that non-specific codes, for example for a painful ankle, were unrelated to the subsequent RA diagnosis. Similarly prescriptions for DMARD such as methotrexate, and especially NSAIDs, may be unrelated to RA. This will lead to an over-estimation of the presenting symptoms and treatment in RA patients. The risk of under-estimation is perhaps greater. This will occur if events or consultations relating to RA diagnosis and management are missed because general codes have been used and the important information recorded in text, which we have described as level 3 variation. The next stage in the project is to extract text around the time of indicator markers, and perform keyword searches on extracted text.

Although we used a panel of four experts for generating the *a priori* lists of indicator markers, a larger panel or a more in depth process, such as a Delphi process, might have generated a different or fuller list. However, other studies in this field have used similar size panels of experts [15] to scrutinise possible codes. Indeed, some studies have not drawn up lists but used a single diagnostic Read code to identify RA patients [34],[35]. We are not aware of any studies which have reported the details of a data-driven stage for modifying their lists. Greater clarity of reporting for drawing up lists would result increased standardisation of case definition and increase the generalisability of EHR study results. Previous studies reporting validation of cases using EHRs have inadequately described the methods for this validation [36]. We believe that this documentation of the process is an important resource for researchers new in the field, and encourage other researchers to publish their code list strategies in a replicable way.

Furthermore we did not look at control data to compare the incidence of the indicator markers in patients with no RA diagnostic code. This comparison would be valuable to ascertain whether these markers do indeed occur in greater numbers just before an RA diagnostic code compared to at other times or in other patients. Further work will examine these markers in control data and also assess their utility in estimating the rates of false negatives which may result by only using diagnostic codes for case definition, thereby introducing bias into the study [28]. The use of control data will allow us to estimate the positive predictive value of clusters of indicator markers to ascertain which combinations are most predictive of a RA diagnostic code being added to the file. However, given the small proportions of patients with three or more of the chosen codes in their record (around 10%) prior to the RA code, there is likely to be more information concealed in the free text which would contribute to case definition. Therefore our plan is to extract this free text information to estimate its contribution to case definition before estimating the predictive value of clusters of indicators.

### Implications for future research and practice

Preparing indicator markers and code-lists is time-consuming and requires input from clinical experts. Context and temporal relationships are often important in looking at markers and building up patterns. It is likely to be a rate-limiting step in future research projects – particularly if multiple clinical entities need to be included in any one study. The design of future EHR systems should consider the need for reporting and aggregation of cases and facilitate the recording of the code-selection process, including a data-driven stage and the ability to search within code-sets. Future research might also consider whether machine learning techniques could contribute to this process.

If EHRs are to be used appropriately in research we need to develop a wider understanding of the drivers of coding in the clinical environment. As work on different diseases develops, it may also be possible to develop a typology of what kind of clinical entities are liable to long gaps between the onset of clinical suspicion, referral and treatment, and where the two are reliably contemporaneous, in order to minimise wasted effort doing over-complex analyses. Our findings here may generalise to some other chronic diseases, but not all.

Sharing of resources between researchers is desirable, to avoid unnecessary duplication. The process of breaking up search strategies into different markers, with publication of precise search terms and results, may allow transparency and replicability in the preparation of code-lists and facilitate their sharing. For example codes relating to a certain presentation might be stored and available for use by another study. At present there is no standardisation of methods for preparing case definitions and code-lists which limits the possibilities of sharing results. No recognised repository to aid sharing exists. Any standardisation would require a common set of meta-data relating to case-finding – detailing what has been done, decisions that have been made and why. The format these meta-data should take is not obvious.

These findings emphasise the need for research using EHR to go beyond simple use of diagnostic codes and adopt more sophisticated strategies for case-finding, including the use of free text. Relying on coded diagnosis may not lead to accurate case definition thereby leading to inaccurate estimates for disease registries and assessments of service needs. The development of automated methods to allow access to information in text without anonymisation should be an urgent priority.

## Supporting Information

Appendix S1 **Searches for indicator markers.**
(DOCX)

Appendix S2 **Final code lists.**
(DOCX)
