# Supplementary material for: Optimising Use of Electronic Health Records to Describe the Presentation of Rheumatoid Arthritis in Primary Care: A Strategy for Developing Code Lists
Source: PLoS One. 2013 Feb 22;8(2):e54878. doi: 10.1371/journal.pone.0054878 (PMC3579840; doi:10.1371/journal.pone.0054878)
Supplement: Appendix S1 — Searches for indicator markers. (DOCX) [file pone.0054878.s001.docx]

# Appendix S1: Searches for indicator markers

1. **Rheumatology monitoring / referral codes**

* Search for rheum referral

*Search_Field Search_Value*

*Read or OXMIS Term *rheumatolog**

* Search for rheum monitoring

*Search_Field Search_Value*

*Read or OXMIS Code 66H**

1. **General arthritis codes (including symptom/sign codes)**

*Search_Field Search_Value*

*Read or OXMIS Code N06**

*Read or OXMIS Code Nz**

*Read or OXMIS Code NY**

*Read or OXMIS Code N09**

*Read or OXMIS Code N04**

1. **Pain codes**

*Search_Field Search_Value*

*Read or OXMIS Code N245**

1. **Stiffness / limitation of movement codes**

*Search_Field Search_Value*

*Read or OXMIS Code 2H**

*Read or OXMIS Code 1D1**

1. **Swelling codes**

*Search_Field Search_Value*

*Read or OXMIS Code 2H3**

1. **Deformity codes**

*Search_Field Search_Value*

*Read or OXMIS Code N362**

*Read or OXMIS Code 2G2**

1. **Rheumatoid Factor test**

*Search_Field Search_Value*

*Read or OXMIS Code 43F**

*Read or OXMIS Term *rheum*fact**

Plus entity type code = 292
