# Supplementary material for: Optimising Use of Electronic Health Records to Describe the Presentation of Rheumatoid Arthritis in Primary Care: A Strategy for Developing Code Lists
Source: PLoS One. 2013 Feb 22;8(2):e54878. doi: 10.1371/journal.pone.0054878 (PMC3579840; doi:10.1371/journal.pone.0054878)
Supplement: Appendix S2 — Final code lists. (DOCX) [file pone.0054878.s002.docx]

**Appendix S2: Final code lists**

Rheumatoid Arthritis Codes

| Readcode | Readterm |
| --- | --- |
| N040.00 | Rheumatoid arthritis |
| N043.00 | Juvenile rheumatoid arthritis - Still's disease |
| N042200 | Rheumatoid nodule |
| N040P00 | Seronegative rheumatoid arthritis |
| N045.00 | Other juvenile arthritis |
| N040T00 | Flare of rheumatoid arthritis |
| N047.00 | Seropositive errosive rheumatoid arthritis |
| H570.00 | Rheumatoid lung |
| N04X.00 | Seropositive rheumatoid arthritis, unspecified |
| 66H..13 | Rheumatoid arthrit. monitoring |
| N040Q00 | Rheumatoid bursitis |
| N040200 | Rheumatoid arthritis of shoulder |
| N043200 | Pauciarticular juvenile rheumatoid arthritis |
| N362200 | Swan-neck finger deformity |
| N041.00 | Felty's syndrome |
| N005.00 | Adult Still's Disease |
| N043z00 | Juvenile rheumatoid arthritis NOS |
| N04..00 | Rheumatoid arthritis and other inflammatory polyarthropathy |
| N04y012 | Fibrosing alveolitis associated with rheumatoid arthritis |
| N040N00 | Rheumatoid vasculitis |
| N040S00 | Rheumatoid arthritis - multiple joint |
| N045100 | Juvenile seronegative polyarthritis |
| F396400 | Myopathy due to rheumatoid arthritis |
| N045500 | Juvenile rheumatoid arthritis |
| N04y000 | Rheumatoid lung |
| N04y200 | Adult-onset Still's disease |
| 2G27.00 | O/E-hands-rheumatoid spindling |
| N043300 | Monarticular juvenile rheumatoid arthritis |
| 2G25.11 | O/E - ulnar deviation |
| N042z00 | Rheumatoid arthropathy + visceral/systemic involvement NOS |
| N040900 | Rheumatoid arthritis of PIP joint of finger |
| N040800 | Rheumatoid arthritis of MCP joint |
| N045000 | Juvenile ankylosing spondylitis |
| G5yA.00 | Rheumatoid carditis |
| N040100 | Other rheumatoid arthritis of spine |
| N040000 | Rheumatoid arthritis of cervical spine |
| N042100 | Rheumatoid lung disease |
| N045600 | Pauciarticular onset juvenile chronic arthritis |
| N043100 | Acute polyarticular juvenile rheumatoid arthritis |
| N040700 | Rheumatoid arthritis of wrist |
| N040B00 | Rheumatoid arthritis of hip |
| N042.00 | Other rheumatoid arthropathy + visceral/systemic involvement |
| G5y8.00 | Rheumatoid myocarditis |
| N043000 | Juvenile rheumatoid arthropathy unspecified |
| N040D00 | Rheumatoid arthritis of knee |
| N040K00 | Rheumatoid arthritis of 1st MTP joint |
| N040F00 | Rheumatoid arthritis of ankle |
| N040R00 | Rheumatoid nodule |
| Nyu1G00 | [X]Seropositive rheumatoid arthritis, unspecified |
| 2G25.00 | O/E - hands - ulnar deviation |
| N04y011 | Caplan's syndrome |
| N040500 | Rheumatoid arthritis of elbow |
| F371200 | Polyneuropathy in rheumatoid arthritis |
| N040A00 | Rheumatoid arthritis of DIP joint of finger |
| N040600 | Rheumatoid arthritis of distal radio-ulnar joint |
| Nyu1200 | [X]Other specified rheumatoid arthritis |
| N040H00 | Rheumatoid arthritis of talonavicular joint |
| N040J00 | Rheumatoid arthritis of other tarsal joint |
| N040G00 | Rheumatoid arthritis of subtalar joint |
| Nyu1100 | [X]Other seropositive rheumatoid arthritis |
| Nyu1500 | [X]Other juvenile arthritis |

Unnamed Inflammatory Arthritis Codes

| **readcode** | **readoxmisterm** |
| --- | --- |
| 7122A | RHEUMATISM PALINDROMIC |
| 715 B | POLYARTHRITIS |
| 7150SN | ARTHRITIS SERO NEGATIVE |
| 7873DM | POLYARTHRALGIA |
| N04..11 | Inflammatory polyarthropathy |
| N045.00 | Other juvenile arthritis |
| N045100 | Juvenile seronegative polyarthritis |
| N04y.00 | Other specified inflammatory polyarthropathy |
| N04y100 | Sero negative arthritis |
| N04y111 | Sero negative polyarthritis |
| N04yz00 | Other specified inflammatory polyarthropathy NOS |
| N04z.00 | Inflammatory polyarthropathy NOS |
| N060.11 | Endemic polyarthritis |
| N062900 | Allergic arthritis of multiple sites |
| N064900 | Transient arthropathy of multiple sites |
| N065.00 | Unspecified polyarthropathy or polyarthritis |
| N065.11 | Polyarthropathy NEC |
| N065900 | Unspecified polyarthropathy of multiple sites |
| N065z00 | Unspecified polyarthropathy or polyarthritis NOS |
| N065z11 | Polyarthritis |
| N06z900 | Arthropathy NOS, of multiple sites |
| N090900 | Effusion of multiple joints |
| N093.00 | Palindromic rheumatism |
| N093.11 | Hench - Rosenberg syndrome |
| N093000 | Palindromic rheumatism of unspecified site |
| N093100 | Palindromic rheumatism of the shoulder region |
| N093200 | Palindromic rheumatism of the upper arm |
| N093300 | Palindromic rheumatism of the forearm |
| N093400 | Palindromic rheumatism of the hand |
| N093500 | Palindromic rheumatism of the pelvic region and thigh |
| N093600 | Palindromic rheumatism of the lower leg |
| N093700 | Palindromic rheumatism of the ankle and foot |
| N093800 | Palindromic rheumatism of other specified site |
| N093900 | Palindromic rheumatism of multiple sites |
| N093z00 | Palindromic rheumatism NOS |
| N094900 | Arthralgia of multiple joints |
| N095900 | Multiple stiff joints |
| Nyu1.00 | [X]Inflammatory polyarthropathies |
| Nyu1500 | [X]Other juvenile arthritis |

Other inflammatory arthritis/condition codes

| readcode | readoxmisterm |
| --- | --- |
| N20..00 | Polymyalgia rheumatica |
| 7149A | RHEUMATIC ARTHRITIS |
| L7873V | ARTHRALGIA VIRAL |
| M160.00 | Psoriatic arthropathy |
| M160.11 |  |
| M160000 | Psoriasis spondylitica |
| M160100 | Distal interphalangeal psoriatic arthropathy |
| M160200 | Arthritis mutilans |
| M160z00 | Psoriatic arthropathy NOS |
| N044.00 | Chronic post-rheumatic arthropathy |
| N044.11 | Jaccoud's syndrome |
| N044.12 | Nodular fibrositis of chronic rheumatic disease |
| N045200 | Juvenile arthritis in psoriasis |
| N045300 | Juvenile arthritis in Crohn's disease |
| N045400 | Juvenile arthritis in ulcerative colitis |
| N067.00 | Ochronotic arthropathy |
| N06y900 | Other specified arthropathy of multiple sites |
| Nyu0.00 | [X]Infectious arthropathies |
| Nyu0000 | [X]Other streptococcal arthritis and polyarthritis |
| Nyu0100 | [X]Arthritis+polyarthritis due/other specfd bacterial agents |
| Nyu0200 | [X]Arthritis in other infectious and parasitic diseases CE |
| Nyu0300 | [X]Other reactive arthropathies |
| Nyu0400 | [X]Other postinfectious arthropathies in diseases CE |
| Nyu0500 | [X]Reactive arthropathy in other diseases CE |
| Nyu1300 | [X]Other psoriatic arthropathies |
| Nyu1400 | [X]Other enteropathic arthropathies |
| Nyu1600 | [X]Juvenile arthritis in other diseases CE |
| Nyu1700 | [X]Other secondary gout |
| Nyu1900 | [X]Other specified crystal arthropathies |
| Nyu1C00 | [X]Gouty arthrpathy due/enzym defects+oth inherit disordr CE |
| Nyu1D00 | [X]Crystal arthropathy in other metabolic disorders CE |
| Nyu1E00 | [X]Arthropathies/oth endocrin,nutritionl+metabolic disorders |
| Nyu2B00 | [X]Other 2ndry arthrosis/1st carpometacarpal joints,bilaterl |
| Nyu2D00 | [X]Other specified arthrosis |
| Nyu4B00 | [X]Arthropathy in hypersensitivity reactions CE |

Synovitis codes

| readcode | readoxmisterm |
| --- | --- |
| N220.00 | Synovitis and tenosynovitis |
| N220000 | Synovitis or tenosynovitis NOS |
| N220Q00 | Transient synovitis |
| N220S00 | Synovitis of hip |
| N220T00 | Synovitis NOS |
| N220V00 | Synovitis of knee |
| N220W00 | Synovitis of elbow |
| N220X00 | Synovitis of shoulder |
| N220z11 | Shoulder synovitis |
| N220z12 | Synovitis of knee |
| N220z13 | Synovitis of elbow |

Non-specific arthritis codes

| **readcode** | **readoxmisterm** |
| --- | --- |
| 66H3.00 | Rheumat.dis.- joints affected |
| 711 A | ARTHRITIS ACUTE NONPYOGENIC |
| 711 AC | ARTHRITIS ACUTE |
| 711 F | ARTHRITIS ACUTE FINGER |
| 711 T | ARTHRITIS ACUTE TOES |
| 7123B | ARTHRITIS ATROPHIC |
| 7149B | ARTHRITIS ALLERGIC |
| 7149C | CLIMACTERIC ARTHRITIS |
| 715 A | ARTHRITIS |
| 715 AG | ARTHRITIS GENERALISED |
| 715 C | ARTHRITIS CHRONIC |
| 715 M | MONOARTHRITIS |
| 715 MR | MONOARTICULAR RHEUMATISM |
| 715 W | WRIST ARTHRITIS |
| 715 WA | ARTHRITIS SHOULDER |
| N06..00 | Other and unspecified arthropathies |
| N062.00 | Allergic arthritis |
| N062000 | Allergic arthritis of unspecified site |
| N062100 | Allergic arthritis of the shoulder region |
| N062200 | Allergic arthritis of the upper arm |
| N062300 | Allergic arthritis of the forearm |
| N062400 | Allergic arthritis of the hand |
| N062500 | Allergic arthritis of the pelvic region and thigh |
| N062600 | Allergic arthritis of the lower leg |
| N062700 | Allergic arthritis of the ankle and foot |
| N062800 | Allergic arthritis of other specified site |
| N062z00 | Allergic arthritis NOS |
| N063.00 | Climacteric arthritis |
| N063.11 | Menopausal arthritis |
| N063000 | Climacteric arthritis of unspecified site |
| N063100 | Climacteric arthritis of the shoulder region |
| N063200 | Climacteric arthritis of the upper arm |
| N063300 | Climacteric arthritis of the forearm |
| N063400 | Climacteric arthritis of the hand |
| N063500 | Climacteric arthritis of the pelvic region and thigh |
| N063600 | Climacteric arthritis of the lower leg |
| N063700 | Climacteric arthritis of the ankle and foot |
| N063800 | Climacteric arthritis of other specified site |
| N063900 | Climacteric arthritis of multiple sites |
| N063z00 | Climacteric arthritis NOS |
| N064.00 | Transient arthropathy |
| N064000 | Transient arthropathy of unspecified site |
| N064100 | Transient arthropathy of the shoulder region |
| N064200 | Transient arthropathy of the upper arm |
| N064300 | Transient arthropathy of the forearm |
| N064400 | Transient arthropathy of the hand |
| N064500 | Transient arthropathy of the pelvic region and thigh |
| N064600 | Transient arthropathy of the lower leg |
| N064700 | Transient arthropathy of the ankle and foot |
| N064800 | Transient arthropathy of other specified site |
| N064A00 | Transient arthropathy of shoulder |
| N064B00 | Transient arthropathy of sternoclavicular joint |
| N064C00 | Transient arthropathy of acromioclavicular joint |
| N064D00 | Transient arthropathy-elbow |
| N064E00 | Transient arthropathy of distal radio-ulnar joint |
| N064F00 | Transient arthropathy-wrist |
| N064G00 | Transient arthropathy of MCP joint |
| N064H00 | Transient arthropathy of PIP joint of finger |
| N064J00 | Transient arthropathy of DIP joint of finger |
| N064K00 | Transient arthropathy-hip |
| N064L00 | Transient arthropathy of sacro-iliac joint |
| N064M00 | Transient arthropathy-knee |
| N064N00 | Transient arthropathy of tibio-fibular joint |
| N064P00 | Transient arthropathy-ankle |
| N064Q00 | Transient arthropathy of subtalar joint |
| N064R00 | Transient arthropathy of talonavicular joint |
| N064S00 | Transient arthropathy of other tarsal joint |
| N064T00 | Transient arthropathy of 1st MTP joint |
| N064U00 | Transient arthropathy of lesser MTP joint |
| N064V00 | Transient arthropathy of IP joint of toe |
| N064z00 | Transient arthropathy NOS |
| N065000 | Unspecified polyarthropathy of unspecified site |
| N065100 | Unspecified polyarthropathy of the shoulder region |
| N065200 | Unspecified polyarthropathy of the upper arm |
| N065300 | Unspecified polyarthropathy of the forearm |
| N065400 | Unspecified polyarthropathy of the hand |
| N065500 | Unspecified polyarthropathy of the pelvic region and thigh |
| N065600 | Unspecified polyarthropathy of the lower leg |
| N065700 | Unspecified polyarthropathy of the ankle and foot |
| N065800 | Unspecified polyarthropathy of other specified site |
| N065A00 | Generalised arthritis |
| N066.00 | Unspecified monoarthritis |
| N066000 | Unspecified monoarthritis of unspecified site |
| N066100 | Unspecified monoarthritis of the shoulder region |
| N066200 | Unspecified monoarthritis of the upper arm |
| N066300 | Unspecified monoarthritis of the forearm |
| N066400 | Unspecified monoarthritis of the hand |
| N066500 | Unspecified monoarthritis of the pelvic region and thigh |
| N066600 | Unspecified monoarthritis of the lower leg |
| N066700 | Unspecified monoarthritis of the ankle and foot |
| N066800 | Unspecified monoarthritis of other specified site |
| N066z00 | Unspecified monoarthritis NOS |
| N069.00 | Arthropathy in neoplastic disease |
| N06y.00 | Other specified arthropathy |
| N06y000 | Other specified arthropathy of unspecified site |
| N06y100 | Other specified arthropathy of the shoulder region |
| N06y200 | Other specified arthropathy of the upper arm |
| N06y300 | Other specified arthropathy of the forearm |
| N06y400 | Other specified arthropathy of the hand |
| N06y500 | Other specified arthropathy of the pelvic region and thigh |
| N06y600 | Other specified arthropathy of the lower leg |
| N06y700 | Other specified arthropathy of the ankle and foot |
| N06y800 | Other specified arthropathy of other specified site |
| N06yz00 | Other specified arthropathy NOS |
| N06z.00 | Arthropathy NOS |
| N06z.11 | Arthritis |
| N06z000 | Arthropathy NOS, of unspecified site |
| N06z100 | Arthropathy NOS, of the shoulder region |
| N06z111 | Shoulder arthritis NOS |
| N06z200 | Arthropathy NOS, of the upper arm |
| N06z211 | Elbow arthritis NOS |
| N06z300 | Arthropathy NOS, of the forearm |
| N06z311 | Wrist arthritis NOS |
| N06z400 | Arthropathy NOS, of the hand |
| N06z411 | Hand arthritis NOS |
| N06z500 | Arthropathy NOS, of the pelvic region and thigh |
| N06z511 | Hip arthritis NOS |
| N06z600 | Arthropathy NOS, of the lower leg |
| N06z611 | Knee arthritis NOS |
| N06z711 | Ankle arthritis NOS |
| N06z712 | Foot arthritis NOS |
| N06z800 | Arthropathy NOS, of other specified site |
| N06zA00 | Acute arthritis |
| N06zB00 | Chronic arthritis |
| N06zz00 | Arthropathy NOS |
| Nyu1A00 | [X]Other specific arthropathies, not elsewhere classified |
| Nyu1B00 | [X]Other specified arthritis |
| Nyu1F00 | [X]Arthropathies in other specified diseases CE |
| Nyu4A00 | [X]Arthropathy in other blood disorders CE |

Other joint symptoms and signs codes

| readcode | readoxmisterm |
| --- | --- |
| 1M10.00 | Knee pain |
| N131.00 | Cervicalgia - pain in neck |
| 1D17.00 | Morning stiffness - joint |
| 2G25.00 | O/E - hands - ulnar deviation |
| 2G25.11 | O/E - ulnar deviation |
| 2G27.00 | O/E-hands-rheumatoid spindling |
| 2H2..00 | O/E - joint abnormal |
| 2H2..11 | O/E - abnormal joint |
| 2H22.00 | O/E - multiple joint abnormal |
| 2H23.00 | O/E - shoulder joint abnormal |
| 2H23.11 | O/E - painful arc |
| 2H24.00 | O/E - elbow joint abnormal |
| 2H25.00 | O/E - wrist joint abnormal |
| 2H26.00 | O/E - hand joint abnormal |
| 2H27.00 | O/E - finger joint abnormal |
| 2H28.00 | O/E - hip joint abnormal |
| 2H29.00 | O/E - knee joint abnormal |
| 2H2A.00 | O/E - ankle joint abnormal |
| 2H2B.00 | O/E - foot joint abnormal |
| 2H2C.00 | O/E - toe joint abnormal |
| 2H2D.00 | O/E - neck joint abnormal |
| 2H2Z.00 | O/E - joint abnormal NOS |
| 2H3..00 | O/E - joint swelling |
| 2H3..11 | O/E - swelling - joint |
| 2H32.00 | O/E - joint effusion present |
| 2H33.00 | O/E -joint synovial thickening |
| 2H3Z.00 | O/E - joint swelling NOS |
| 2H42.00 | O/E - abnormal joint movement |
| 2H43.00 | O/E - reduced joint movement |
| 2H43000 | O/E - reduced movement of spine |
| 2H43100 | O/E - reduced movement of arm |
| 2H43200 | O/E - reduced movement of wrist |
| 2H43300 | O/E - reduced movement of hip |
| 2H44.00 | O/E-joint-passive>active movet |
| 2H45.00 | O/E - joint movement painful |
| 2H46.00 | O/E - joint crepitation |
| 2H52.00 | O/E - fixed joint deformity |
| 2H53.00 | O/E - unfixed joint deformity |
| 2H54.00 | O/E - joint unstable |
| 2H6..00 | O/E - joint stiffness |
| 2H62.00 | O/E - joint stiff |
| 2H6Z.00 | O/E - joint stiffness NOS |
| 2H8..00 | O/E - spine abnormal |
| 2H82.00 | O/E - cervical spine abnormal |
| 2H83.00 | O/E - thoracic spine abnormal |
| 2H84.00 | O/E - lumbar spine abnormal |
| 2H85.00 | O/E - sacrum abnormal |
| 2H86.00 | O/E - coccyx abnormal |
| 2H8Z.00 | O/E - spine abnormal NOS |
| 2HB8.00 | O/E - bone - crepitus |
| 2HC..11 | O/E - soft tissue swelling |
| 2HC4.00 | O/E - soft tissue crepitus |
| 66H4.00 | Rheumat. symptom change |
| 710 JI | INFLAMMATION JOINT |
| 7130PP | PIP JOINT LUMPS |
| 7270A | MOTION LIMITED BACK |
| 7270AP | SPINE RESTRICTED MOVEMENT |
| 7271B | STIFF SHOULDER |
| 7271D | SHOULDER LIMITATION MOVEMENT PASSIVE |
| 7272 | STIFF ELBOW |
| 7272A | MOVEMENT LIMITATION PASSIVE ARM |
| 7272B | STIFF ARM |
| 7273A | WRIST LIMITATION MOVEMENT |
| 7273B | WRIST STIFF |
| 7274B | STIFF FINGER |
| 7275A | MOVEMENT LIMITATION HIP |
| 7275B | STIFF HIP |
| 7275D | MOVEMENT LIMITATION PASSIVE HIP |
| 7276B | STIFF KNEE |
| 7276D | MOVEMENT LIMITATION PASSIVE KNEE JOINT |
| 7277A | MOVEMENT LIMITATION PASSIVE ANKLE |
| 7277B | STIFF ANKLE |
| 7279B | STIFF JOINT |
| 729 B | JOINT HYDRARTHROSIS |
| 729 BA | EFFUSION JOINT ACUTE |
| 729 BC | EFFUSION JOINT CHRONIC |
| 729 BF | FLUID JOINT |
| 729 BN | INTERMITTENT HYDRARTHROSIS |
| 729 C | FLUID KNEE |
| 729 FH | CHRONIC INFLAMMATION SHOULDER |
| 731 EB | SWELLING ELBOW |
| 7871KH | ANKLE ACHE |
| 7871KP | PAIN ANKLE |
| 7871KR | SORE ANKLE |
| 7871PH | ACHE THUMB |
| 7871TH | ACHE TOE |
| 7873A | PAIN JOINT |
| 7873AH | JOINT ACHE |
| 7873AL | ARTHRALGIA |
| 7873AP | PAIN PERIARTICULAR |
| 7873AR | SORE JOINT |
| 7873B | PAIN ELBOW |
| 7873BH | ACHE ELBOW |
| 7873C | PAIN HIP |
| 7873CH | ACHE HIP |
| 7873DH | ACHE JOINTS |
| 7873E | PAIN KNEE |
| 7873EH | ACHE KNEE |
| 7873F | SHOULDER PAIN |
| 7873FH | ACHE SHOULDER |
| 7873G | PAIN WRIST |
| 7873GH | ACHE WRIST |
| 7873PT | PAIN PELVIS JOINT |
| 7874 | JOINT SWELLING |
| 7874A | SWELLING ANKLE JOINT |
| 7874B | SWELLING KNEE |
| 7874FP | SWELLING PROXIMAL INTERPHALANGEAL JOINTS |
| 7874HP | SWELLING HIP |
| 7874M | SWELLING JOINTS |
| L5199C | CREPITUS |
| N066.11 | Coxitis |
| N06z700 | Arthropathy NOS, of the ankle and foot |
| N090.00 | Effusion of joint |
| N090.11 | Hydrarthrosis |
| N090.12 | Swelling of joint - effusion |
| N090000 | Joint effusion of unspecified site |
| N090100 | Joint effusion of the shoulder region |
| N090200 | Joint effusion of the upper arm |
| N090211 | Elbow joint effusion |
| N090300 | Joint effusion of the forearm |
| N090311 | Wrist joint effusion |
| N090400 | Joint effusion of the hand |
| N090500 | Joint effusion of the pelvic region and thigh |
| N090511 | Hip joint effusion |
| N090600 | Joint effusion of the lower leg |
| N090611 | Knee joint effusion |
| N090700 | Joint effusion of the ankle and foot |
| N090711 | Ankle joint effusion |
| N090800 | Joint effusion of other specified site |
| N090A00 | Effusion of shoulder |
| N090B00 | Effusion of sternoclavicular joint |
| N090C00 | Effusion of acromioclavicular joint |
| N090D00 | Effusion of elbow |
| N090E00 | Effusion of distal radio-ulnar joint |
| N090F00 | Effusion of wrist |
| N090G00 | Effusion of MCP joint |
| N090H00 | Effusion of PIP joint of finger |
| N090J00 | Effusion of DIP joint - finger |
| N090K00 | Effusion of hip |
| N090L00 | Effusion of sacro-iliac joint |
| N090M00 | Effusion of knee |
| N090N00 | Effusion of tibio-fibular joint |
| N090P00 | Effusion of ankle |
| N090Q00 | Effusion of subtalar joint |
| N090R00 | Effusion of talonavicular joint |
| N090S00 | Effusion of other tarsal joint |
| N090T00 | Effusion of 1st MTP joint |
| N090U00 | Effusion of lesser MTP joint |
| N090V00 | Effusion of IP joint of toe |
| N090W00 | Intermittent hydrarthrosis |
| N090X00 | Chronic joint effusion |
| N090Y00 | Acute joint effusion |
| N090z00 | Effusion of joint NOS |
| N093.12 | Intermittent hydrarthrosis |
| N093.13 | Intermittent joint effusion |
| N094.00 | Pain in joint - arthralgia |
| N094.11 | Ache in joint |
| N094000 | Arthralgia of unspecified site |
| N094100 | Arthralgia of the shoulder region |
| N094111 | Shoulder joint pain |
| N094200 | Arthralgia of the upper arm |
| N094211 | Elbow joint pain |
| N094300 | Arthralgia of the forearm |
| N094311 | Wrist joint pain |
| N094400 | Arthralgia of the hand |
| N094411 | Hand joint pain |
| N094500 | Arthralgia of the pelvic region and thigh |
| N094511 | Coxalgia |
| N094512 | Hip joint pain |
| N094600 | Arthralgia of the lower leg |
| N094611 | Knee joint pain |
| N094700 | Arthralgia of the ankle and foot |
| N094711 | Ankle joint pain |
| N094800 | Arthralgia of other specified site |
| N094A00 | Arthralgia of shoulder |
| N094B00 | Arthralgia of sternoclavicular joint |
| N094C00 | Arthralgia of acromioclavicular joint |
| N094D00 | Arthralgia of elbow |
| N094D11 | Elbow joint pain |
| N094E00 | Arthralgia of distal radio-ulnar joint |
| N094F00 | Arthralgia of wrist |
| N094G00 | Arthralgia of MCP joint |
| N094H00 | Arthralgia of PIP joint of finger |
| N094J00 | Arthralgia of DIP joint of finger |
| N094K00 | Arthralgia of hip |
| N094K11 | Coxalgia |
| N094K12 | Hip pain |
| N094L00 | Arthralgia of sacro-iliac joint |
| N094M00 | Arthralgia of knee |
| N094N00 | Arthralgia of tibio-fibular joint |
| N094P00 | Arthralgia of ankle |
| N094Q00 | Arthralgia of subtalar joint |
| N094R00 | Arthralgia of talonavicular joint |
| N094S00 | Arthralgia of other tarsal joint |
| N094T00 | Arthralgia of 1st MTP joint |
| N094U00 | Arthralgia of lesser MTP joint |
| N094V00 | Arthralgia of IP joint of toe |
| N094W00 | Anterior knee pain |
| N094z00 | Arthralgia NOS |
| N095.00 | Joint stiffness NEC |
| N095000 | Stiff joint NEC, of unspecified site |
| N095100 | Stiff joint NEC, of the shoulder region |
| N095111 | Shoulder stiff |
| N095200 | Stiff joint NEC, of the upper arm |
| N095211 | Elbow stiff |
| N095300 | Stiff joint NEC, of the forearm |
| N095311 | Wrist stiff |
| N095400 | Stiff joint NEC, of the hand |
| N095411 | Hand joint stiff |
| N095500 | Stiff joint NEC, of the pelvic region and thigh |
| N095511 | Hip stiff |
| N095600 | Stiff joint NEC, of the lower leg |
| N095611 | Knee stiff |
| N095700 | Stiff joint NEC, of the ankle and foot |
| N095711 | Ankle stiff |
| N095800 | Stiff joint NEC, of other specified site |
| N095A00 | Stiff shoulder NEC |
| N095B00 | Stiff sternoclavicular joint NEC |
| N095C00 | Stiff acromioclavicular joint NEC |
| N095D00 | Stiff elbow NEC |
| N095E00 | Stiff distal radio-ulnar joint NEC |
| N095F00 | Stiff wrist NEC |
| N095G00 | Stiff MCP joint NEC |
| N095H00 | Stiff PIP joint of finger NEC |
| N095J00 | Stiff DIP joint of finger NEC |
| N095K00 | Stiff hip NEC |
| N095L00 | Stiff sacro-iliac joint NEC |
| N095M00 | Stiff knee NEC |
| N095N00 | Stiff tibio-fibular joint NEC |
| N095P00 | Stiff ankle NEC |
| N095Q00 | Stiff subtalar joint NEC |
| N095R00 | Stiff talonavicular joint NEC |
| N095S00 | Stiff other tarsal joint NEC |
| N095T00 | Stiff 1st MTP joint NEC |
| N095U00 | Stiff lesser MTP joint NEC |
| N095V00 | Stiff IP joint of toe NEC |
| N095W00 | Stiff finger |
| N095z00 | Joint stiffness NEC, NOS |
| N096.00 | Other joint symptoms |
| N096.11 | Joint crepitus |
| N096000 | Other joint symptoms of unspecified site |
| N096100 | Other joint symptoms of the shoulder region |
| N096200 | Other joint symptoms of the upper arm |
| N096300 | Other joint symptoms of the forearm |
| N096400 | Other joint symptoms of the hand |
| N096500 | Other joint symptoms of the pelvic region and thigh |
| N096600 | Other joint symptoms of the lower leg |
| N096700 | Other joint symptoms of the ankle and foot |
| N096800 | Other joint symptoms of other specified site |
| N096900 | Other joint symptoms of multiple sites |
| N245.11 | Ankle pain |
| N245.14 | Hand pain |
| N245.17 | Shoulder pain |
| N245000 | Hand pain |
| N245700 | Shoulder pain |
| N362100 | Boutonniere finger deformity |
| N362200 | Swan-neck finger deformity |
| n094200 | Arthralgia of the upper arm |

Rheumatoid factor test codes

| readcode | readoxmisterm |
| --- | --- |
| 43F..00 | Rheumatoid factor |
| 43F..11 | Latex test |
| 43F..12 | Rose Waaler test |
| 43F1.00 | Rheumatoid factor positive |
| 43F2.00 | Rheumatoid factor negative |
| 43F3.00 | R.A. latex test |
| 43F4.00 | Rose Waaler test - sheep cells |
| 43F4000 | Heterophile agglutin test normal |
| 43F4100 | Heterophile agglutin test abnormal |
| 43F5.00 | Serum rheumatoid antigen level |
| 43F6.00 | Fluid rheumatoid factor level |
| 43F7. | Rheumatoid factor screening test |
| 43F7.00 | Rheumatoid factor screening test |
| 43F8.00 | Serum rheumatoid antibody level |
| 43F9.00 | Rheumatoid factor IgG level |
| 43FA.00 | Rheumatoid factor IgM level |
| 43FB.00 | IgA rheumatoid factor level |
| 43FZ.00 | Rheumatoid factor NOS |
| 43b9.00 | Rheumatoid arthritis particle agglutination test |
| 43c6.00 | Rheumatoid arthritis screening test |
| 68F1.00 | Rheumatoid arthritis screen |
| L 150C | HETEROPHILE AGGLUTIN TEST NORMAL |
| L 150CA | HETEROPHILE AGGLUTIN TEST |
| L 150D | HETEROPHILE AGGLUTIN TEST ABNORMAL |
| L 151A | ROSE WAALER TEST NEGATIVE |
| L 151AA | ROSE WAALER TEST |
| L 151B | ROSE WAALER TEST POSITIVE |
| L 151C | RAHA TEST NEGATIVE |
| L 151CA | RAHA TEST |
| L 151D | RAHA TEST POSITIVE |
| L 151EA | RHEUMATOID FACTOR |
| L1510A | RA SCREEN |
| L1510N | RA SCREEN NEGATIVE |
| L1510P | RA SCREEN POSITIVE |
| ZV7y100 | [V]Screening for rheumatoid arthritis |

Referral to Rheumatology Codes

| readcode | readoxmisterm |
| --- | --- |
| 66H..00 | Rheumatol. disorder monitoring |
| 66H..11 | Arthritis monitoring |
| 66H..12 | Rheumatism monitoring |
| 66H1.00 | Rheumat. initial assessment |
| 66H2.00 | Rheumat. follow-up assessment |
| 66H5.00 | Rheumat. drug side effect |
| 66H6.00 | Rheumat. treatment change |
| 66H6.11 | Rheumat.dis.treatment changed |
| 66H7.00 | Rheumat.dis.treatment started |
| 66H8.00 | Rheumat.dis.treatment stopped |
| 66H9.00 | Rheumatology management plan given |
| 66HZ.00 | Rheumatol.dis. monitoring NOS |
| 679M.00 | Health education - rheumatology |
| 67Ih.00 | Advice to GP from rheumatology service |
| 8H2C.00 | Admit rheumatology emergency |
| 8H3H.00 | Non-urgent rheumatology admisn |
| 8H4B.00 | Referred to rheumatologist |
| 8HJC.00 | Rheumatology self-referral |
| 8HKA.00 | Rheumatology D.V. requested |
| 8HLA.00 | Rheumatology D.V. done |
| 8HMA.00 | Listed for Rheumatology admiss |
| 8HTd.00 | Referral to rheumatology clinic |
| 8HVQ.00 | Private referral to rheumatologist |
| 9N1O.00 | Seen in rheumatology clinic |
| 9b9M.00 | Rheumatology |
| L0010AW | SEEN IN RHEUMATOLOGY CLINIC |
| L0010EW | REFERRED TO RHEUMATOLOGY CLINIC |
| ZL18T00 | Under care of rheumatologist |
| ZL22G00 | Under care of rheumatology nurse specialist |
| ZL5AR00 | Referral to rheumatologist |
| ZL62G00 | Referral to rheumatology nurse specialist |
| ZL9AT00 | Seen by rheumatologist |
| ZLA2G00 | Seen by rheumatology nurse specialist |
| ZLD3T00 | Discharge by rheumatologist |
| ZLD7E00 | Discharge by rheumatology nurse specialist |
| ZLE6Q00 | Discharge from rheumatology service |

DMARD codes

| prodcode | productname |
| --- | --- |
| 265 | chloroquine sulphate syrup 68mg/5ml |
| 267 | penicillamine tablets 50mg |
| 270 | azathioprine injection 50mg/vial |
| 283 | MYOCRISIN injection 10mg/0.5ml [SANOFI/AVE] |
| 380 | SALAZOPYRIN EN- tablets 500mg [PHARMACIA] |
| 422 | chloroquine phosphate tablets 250mg |
| 451 | azathioprine tablets 25mg |
| 456 | chloroquine sulphate tablets 200mg |
| 463 | NIVAQUINE injection 272.5mg(200mg base)/5ml [AVENTIS] |
| 508 | sulfasalazine enteric coated tablets 500mg |
| 571 | azathioprine tablets 50mg |
| 604 | penicillamine tablets 250mg |
| 643 | penicillamine tablets 125mg |
| 671 | IMURAN tablets 25mg [WELLCOME] |
| 672 | hydroxychloroquine sulphate tablets 200mg |
| 770 | azathioprine capsules |
| 823 | methotrexate tablets 2.5mg |
| 877 | methotrexate tablets 10mg |
| 972 | NEORAL capsules 25mg [NOVARTIS] |
| 973 | NEORAL capsules 100mg [NOVARTIS] |
| 1566 | SALAZOPYRIN tablets 500mg [PHARMACIA] |
| 1626 | ciclosporin oral solution 100mg/ml |
| 1899 | IMURAN tablets 50mg [WELLCOME] |
| 1905 | NEORAL oral solution 100mg/ml [NOVARTIS] |
| 2837 | ciclosporin capsules 50mg |
| 2838 | ciclosporin capsules 25mg |
| 2839 | tacrolimus twice daily capsules 1mg |
| 2920 | sulfasalazine tablets 500mg |
| 3169 | NIVAQUINE syrup 68mg/5ml [AVENTIS] |
| 3224 | NIVAQUINE tablets 200mg [AVENTIS] |
| 3267 | MYOCRISIN injection 50mg/0.5ml [SANOFI/AVE] |
| 3325 | AVLOCLOR tablets 250mg [ASTRAZENEC] |
| 3327 | DISTAMINE tablets 125mg [ALLIANCE] |
| 3329 | MYOCRISIN injection 20mg/0.5ml [SANOFI/AVE] |
| 3683 | PROGRAF twice daily capsules 1mg [ASTELLAS] |
| 3896 | ciclosporin capsules 100mg |
| 3920 | SANDIMMUN capsules 25mg [NOV/SANDOZ] |
| 3934 | auranofin tablets 3mg |
| 4230 | mycophenolate mofetil tablets 500mg |
| 4231 | NEORAL capsules 50mg [NOVARTIS] |
| 4438 | mycophenolate mofetil capsules 250mg |
| 4470 | sodium aurothiomalate injection 50mg/0.5ml |
| 4946 | PLAQUENIL tablets 200mg [SANOFI S] |
| 4970 | leflunomide tablets 100mg |
| 4971 | leflunomide tablets 10mg |
| 4978 | SALAZOPYRIN tablets 500mg [PHARMACIA] |
| 5089 | tacrolimus twice daily capsules 5mg |
| 5427 | sulfasalazine tablets 500mg |
| 5870 | PROGRAF twice daily capsules 500 micrograms [ASTELLAS] |
| 6484 | sirolimus tablets 2mg |
| 6495 | tacrolimus twice daily capsules 500 micrograms |
| 6600 | sirolimus tablets 1mg |
| 6882 | adalimumab injection 40mg |
| 6934 | leflunomide tablets 20mg |
| 7077 | mycophenolate mofetil powder for concentrate for solution for infusion 500mg |
| 7336 | methotrexate injection 12.5mg/0.5ml |
| 7337 | methotrexate injection 10mg/0.4ml |
| 7497 | sulfasalazine suspension 250mg/5ml |
| 8327 | methotrexate injection 50mg/3ml |
| 8583 | methotrexate injection 25mg/ml |
| 8904 | DISTAMINE tablets 250mg [ALLIANCE] |
| 9528 | methotrexate injection 5mg/2ml |
| 10658 | chloroquine phosphate syrup 80mg/5ml |
| 10842 | sodium aurothiomalate injection 10mg/0.5ml |
| 11767 | sulfasalazine suspension 250mg/5ml |
| 11959 | DISTAMINE tablets 50mg [ALLIANCE] |
| 12339 | AZAMUNE tablets 50mg [PENN] |
| 12816 | methotrexate injection 100mg/ml |
| 13022 | NIVAQUINE tablets 200mg [BEACON] |
| 13271 | PROGRAF twice daily capsules 5mg [ASTELLAS] |
| 13320 | azathioprine tablets 10mg |
| 13428 | MAXTREX tablets 2.5mg [PHARMACIA] |
| 13493 | RIDAURA TILTAB tablets 3mg [ASTELLAS] |
| 13494 | SANDIMMUN sugar free solution 100mg/ml [NOV/SANDOZ] |
| 13556 | SANDIMMUN capsules 100mg [NOV/SANDOZ] |
| 14054 | SALAZOPYRIN suspension 250mg/5ml [PHARMACIA] |
| 14347 | methotrexate injection 20mg/0.8ml |
| 14348 | METOJECT injection 20mg/2ml [MEDAC UK] |
| 14395 | IMURAN injection 50mg/vial [WELLCOME] |
| 14748 | methotrexate sodium injection 25mg/ml |
| 14828 | NIVAQUINE syrup 68mg/5ml [SANOFI/AVE] |
| 14886 | ENBREL powder for solution for injection 25mg [WYETH PHAR] |
| 15362 | chloroquine sulphate injection 272.5mg(200mg base)/5ml |
| 15596 | SANDIMMUN capsules 50mg [NOV/SANDOZ] |
| 15921 | etanercept powder for solution for injection 25mg |
| 16035 | ciclosporin capsules 10mg |
| 16137 | NEORAL capsules 10mg [NOVARTIS] |
| 16519 | methotrexate injection 25mg/1ml |
| 16522 | ARAVA tablets 10mg [AVENTIS] |
| 16540 | methotrexate injection 15mg/0.6ml |
| 16570 | methotrexate injection 7.5mg/0.3ml |
| 16606 | sodium aurothiomalate injection 20mg/0.5ml |
| 16822 | infliximab powder for concentrate for solution for infusion 100mg |
| 16879 | mycophenolate mofetil oral suspension 1g/5ml |
| 16919 | CELLCEPT capsules 250mg [ROCHE] |
| 17035 | methotrexate suspension 2.5mg/5ml |
| 17410 | deflazacort tablets 30mg |
| 17642 | ARAVA tablets 20mg [AVENTIS] |
| 17672 | methotrexate injection 22.5mg/0.9ml |
| 17880 | SALAZOPYRIN suspension 250mg/5ml [PHARMACIA] |
| 18424 | methotrexate sodium tablets 2.5mg |
| 18460 | ARAVA tablets 100mg [AVENTIS] |
| 18804 | CELLCEPT tablets 500mg [ROCHE] |
| 18890 | methotrexate injection 17.5mg/0.7ml |
| 19072 | OPRISINE tablets 50mg [OPUS] |
| 19257 | ENBREL powder for solution for injection 50mg [WYETH PHAR] |
| 19370 | ciclosporin concentrate for solution for infusion 50mg/ml |
| 20097 | sirolimus oral solution 1mg/ml |
| 20255 | PENDRAMINE tablets 250mg [VIATRIS] |
| 20862 | SULFASALAZINE enteric coated tablets 500mg [ACTAVIS] |
| 20951 | METHOTREXATE tablets 2.5mg [GOLDSHIELD] |
| 21753 | MAXTREX tablets 10mg [PHARMACIA] |
| 21899 | IMMUNOPRIN tablets 50mg [ASHBOURNE] |
| 22392 | REMICADE powder for concentrate for solution for infusion 100mg [SCHERING-P] |
| 22982 | azathioprine oral solution 50mg/5ml |
| 23289 | RAPAMUNE tablets 1mg [WYETH PHAR] |
| 23401 | SULAZINE EC enteric coated tablets 500mg [CHATFIELD] |
| 23441 | MALARIVON syrup 80mg/5ml [WALLACE] |
| 23850 | HUMIRA injection 40mg [ABBOTT] |
| 24634 | methotrexate injection 25mg/2.5ml |
| 24783 | methotrexate injection 50mg/2ml |
| 26064 | methotrexate injection 20mg/2ml |
| 26097 | mycophenolic acid gastro-resistant tablets 360mg |
| 26261 | BERKAPRINE tablets 50mg [RORER] |
| 26387 | etanercept powder for solution for injection 50mg |
| 26790 | SANDIMMUN concentrate for solution for infusion 50mg/ml [NOV/SANDOZ] |
| 27289 | MYFORTIC tablets 360mg [NOVARTIS] |
| 27290 | MYFORTIC tablets 180mg [NOVARTIS] |
| 27342 | MAXTREX injection 2.5mg/ml [PHARMACIA] |
| 27400 | METOJECT injection 15mg/1.5ml [MEDAC UK] |
| 27404 | methotrexate injection 15mg/1.5ml |
| 27642 | methotrexate injection 27.5mg/1.1ml |
| 28041 | methotrexate oral suspension 12.5mg/5ml |
| 28490 | rituximab concentrate for solution for infusion 100mg/10ml |
| 28999 | RAPAMUNE tablets 2mg [WYETH PHAR] |
| 29069 | methotrexate sterile powder 500mg/vial |
| 29340 | AZATHIOPRINE tablets 50mg [IVAX] |
| 29566 | hydroxychloroquine sulphate oral solution 200mg/5ml |
| 29721 | PENDRAMINE tablets 125mg [VIATRIS] |
| 30495 | IMURAN tablets 10mg [WELLCOME] |
| 30703 | methotrexate injection 30mg/1.2ml |
| 30780 | METHOTREXATE tablets 2.5mg [PHARMACIA] |
| 30925 | PENICILLAMINE tablets 250mg [ACTAVIS] |
| 30932 | methotrexate injection 5mg/0.2ml |
| 31120 | PENICILLAMINE tablets 125mg [IVAX] |
| 31215 | AZATHIOPRINE tablets 50mg [KENT] |
| 31216 | PENICILLAMINE tablets 125mg [ACTAVIS] |
| 31217 | PENICILLAMINE tablets 250mg [HILLCROSS] |
| 31667 | SULFASALAZINE tablets 500mg [HILLCROSS] |
| 31949 | SULFASALAZINE tablets 500mg [ACTAVIS] |
| 32101 | AZATHIOPRINE tablets 25mg [HILLCROSS] |
| 32111 | METHOTREXATE tablets 2.5mg [MAYNE] |
| 32229 | methotrexate injection 500mg/20ml |
| 32418 | KINERET injection 100mg/0.67ml [AMGEN] |
| 32614 | SIMULECT powder for solution for infusion 20mg [NOVARTIS] |
| 32865 | methotrexate injection 10mg/1ml |
| 33123 | tacrolimus concentrate for solution for infusion 5mg/1ml |
| 33601 | METOJECT injection 25mg/2.5ml [MEDAC UK] |
| 33682 | SULFASALAZINE enteric coated tablets 500mg [DDSA] |
| 33728 | RAPAMUNE oral solution 1mg/ml [WYETH PHAR] |
| 33968 | SULFASALAZINE tablets 500mg [APS] |
| 34258 | METHOTREXATE injection 20mg/0.8ml [CENT HOME] |
| 34451 | AZATHIOPRINE tablets 50mg [GEN (UK)] |
| 34473 | SULFASALAZINE tablets 500mg [GEN (UK)] |
| 34684 | PENICILLAMINE tablets 250mg [GEN (UK)] |
| 34687 | AZATHIOPRINE tablets 50mg [HILLCROSS] |
| 34816 | AZATHIOPRINE tablets 25mg [GEN (UK)] |
| 34894 | SULFASALAZINE enteric coated tablets 500mg [CERETRON] |
| 34929 | METHOTREXATE tablets 10mg [MAYNE] |
| 35126 | etanercept injection solution 50mg |
| 35301 | mycophenolic acid gastro-resistant tablets 180mg |
| 35402 | methotrexate injection 7.5mg/0.75ml |
| 35419 | ENBREL injection solution 25mg [WYETH PHAR] |
| 35518 | azathioprine oral suspension 50mg/5ml |
| 35752 | methotrexate oral suspension 7.5mg/5ml |
| 35865 | METOJECT injection 7.5mg/0.75ml [MEDAC UK] |
| 36008 | etanercept injection solution 25mg |
| 36167 | methotrexate injection 1000mg/10ml |
| 36294 | rituximab concentrate for solution for infusion 500mg/50ml |
| 36556 | ENBREL injection solution 50mg [WYETH PHAR] |
| 36726 | anakinra injection 100mg/0.67ml |
| 36792 | azathioprine oral solution 50mg/ml |
| 36800 | methotrexate oral solution 10mg/5ml |
| 36849 | methotrexate oral suspension 10mg/5ml |
| 37117 | METOJECT injection 10mg/1ml [MEDAC UK] |
| 37155 | tacrolimus suspension 1mg/ml |
| 37506 | ADVAGRAF once daily modified release capsules 1mg [ASTELLAS] |
| 37915 | basiliximab powder for solution for infusion 10mg |
| 37985 | tacrolimus once daily modified release capsules 1mg |
| 38056 | ciclosporin concentrate for solution for infusion 50mg/1ml |
| 38113 | tacrolimus once daily modified release capsules 500 micrograms |
| 38919 | ADVAGRAF once daily modified release capsules 5mg [ASTELLAS] |
| 39111 | rituximab concentrate for intravenous infusion 10mg/ml |
| 39115 | azathioprine capsules 10mg |
| 39633 | ADVAGRAF once daily modified release capsules 500 micrograms [ASTELLAS] |
| 40170 | PENICILLAMINE tablets 125mg [GEN (UK)] |

Non-specific auto-immune investigation codes

| readcode | readoxmisterm |
| --- | --- |
| 43G1.00 | Anti-nuclear factor |
| 43G1.11 | Anti-nuclear antibody |
| 43G2.00 | Antimitochondrial autoantibod. |
| 43G3.00 | Anti smooth muscle autoantibod |
| 43G4.00 | Parietal cell autoantibodies |
| 43GG.00 | Autoimmune profile |
| 43GW.00 | Anti liver kidney microsomal antibody level |
| 44CC.00 | Plasma C reactive protein |
| 44CS.00 | Serum C reactive protein level |
